# Durability of Ultra‐Low Temperature Cryoablation Lesions in Atrial Fibrillation: Insights From Repeat Ablation Procedures

**DOI:** 10.1111/jce.16665

**Published:** 2025-03-27

**Authors:** Bob G. S. Abeln, Lucio Addeo, Tom De Potter, Lucas V. A. Boersma

**Affiliations:** ^1^ Department of Cardiology St. Antonius Hospital Nieuwegein the Netherlands; ^2^ Department of Cardiology Amsterdam University Medical Centers Amsterdam the Netherlands; ^3^ Department of Cardiology Onze Lieve Vrouw Hospital Aalst Belgium

**Keywords:** ablation lesion durability, atrial fibrillation, posterior wall isolation, pulmonary vein isolation, repeat ablation, ultra‐low temperature cryoablation

## Abstract

**Background:**

Ultra‐low temperature cryoablation (ULTC) is a technique designed to rapidly cool cardiac tissue to extremely low temperatures, enabling the creation of ablation lesions for the treatment of atrial fibrillation (AF). Prior studies have demonstrated low rates of arrhythmia recurrence, but little is known about ablation lesion durability.

**Methods:**

Patients undergoing repeat ablation were selected from the CryoCure2 (NCT02839304) and iCLAS PMCF(NCT05416086) studies. Baseline patient and ULTC procedure characteristics were evaluated. During repeat ablation, ULTC ablation lesions were assessed for electrical block, including segment‐based assessment of pulmonary vein (PV) ablation lesions. Arrhythmia outcomes after repeat ablation were evaluated.

**Results:**

Twenty‐five patients were included in the cohort: Age 68 ± 7 years, male 68%, persistent AF 68%, LAVI 42 ± 24 mL/m^2^. During index procedure, ULTC was used to target the PVs in all patients, the left atrium posterior wall (LAPW) in 15 patients, the lateral mitral isthmus (LMI) in five patients and the cavotricuspid isthmus (CTI) in two patients. At repeat ablation, PV reconnection was observed in 21/25 patients (55/100 PVs reconnected), and reconnection occurred most often in the anterior segments of the left PVs. The LAPW lesion was incomplete in 4/15 patients, the LMI in 3/5 and the CTI in 1/2. After repeat ablation, 10/25 patients had arrhythmia recurrence.

**Conclusion:**

Reconnection of ablation targets during repeat ablation for arrhythmia recurrence following ULTC occurred at rates comparable to those observed with conventional thermal ablation modalities. The anterior side of the left PVs appears to be reconnected most often.

**Abbreviations**
AF: Atrial fibrillation
CBA: Cryoballoon ablation
CTI: Cavotricuspid isthmus
LAPW: Left atrium posterior wall
LMI: Lateral mitral isthmus
PV: Pulmonary vein
PVI: Pulmonary vein isolation
RFA: Radiofrequency ablation
ULTC: Ultra‐low temperature cryoablation

## 1 Introduction

Pulmonary vein (PV) isolation (PVI) is the cornerstone of catheter ablation for atrial fibrillation (AF). Conventional thermal ablation modalities, such as radiofrequency (RFA) and cryoballoon (CBA) ablation, are capable of achieving acute electrical isolation in the majority of patients. However, despite acute success, 35%–49% of patients have recurrence of atrial tachyarrhythmia at 1‐year follow‐up [1, 2]. In prior studies which assessed repeat ablation procedures to treat arrhythmia recurrence, electrical reconnection was observed in up to 67% of PVs and 69%–100% of patients had at least one PV that was not durably isolated [3, 4]. As PVI is the cornerstone of catheter ablation of AF and PV reconnection is observed often in patients with arrhythmia recurrence, lesion durability could be an important contributor to long‐term procedural success.

More recently, ultra‐low temperature cryoablation (ULTC) was introduced as a novel ablation energy modality. This modality cools the catheter using “near‐critical” nitrogen, which theoretically enables minimum temperatures as low as—196°C [5]. First clinical studies have acute procedural success rates that are similar to conventional thermal ablation modalities and only 14% recurrence of AF at 12‐months follow‐up [6]. These rates of procedural success may be attributed to the ablation lesion characteristics of ULTC, that have been shown to be contiguous, transmural and durable in preclinical studies [7]. However, to date no clinical studies have investigated the mechanism of arrhythmia recurrence after ULTC.

In this paper, we describe a cohort of patients that had a repeat ablation procedure to treat arrhythmia recurrence after ULTC for AF. We evaluated baseline and procedural characteristics, the pattern of reconnections, and arrhythmia recurrence after repeat ablation.

## 2 Methods

### 2.1 Study Population

The population for this investigation was derived from two prospective, multicenter, observational studies in which the iCLAS Cryoablation System (Adagio Medical, Laguna Hills, USA) was used to perform PVI to treat AF: the CryoCure‐2 study (ClinicalTrials. gov Identifier: NCT02839304) and the iCLAS Cryoablation System PMCF study (NCT05416086). The present study included all patients that had a repeat ablation procedure to treat arrhythmia recurrence after ULTC using the AF4 ablation catheter, between February 2019 and February 2024 at the OLV Hospital (Aalst, Belgium) and the St. Antonius Hospital (Nieuwegein, The Netherlands). The study complied with the Declaration of Helsinki, research protocols were approved by the local ethics committee, and all subjects provided informed consent.

### 2.2 Ultra‐Low Temperature Cryoablation

**Figure 1:**
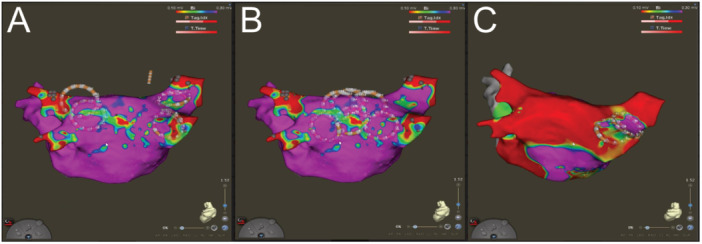
Ablation of various targets using ULTC, guided by an electroanatomic mapping system (A) Pulmonary vein isolation using the circular catheter configuration at the pulmonary vein antrum; (B) Left atrium posterior wall isolation using overlapping circular ablation lesions between the pulmonary veins; (C) Additional applications at the right superior pulmonary vein after successful ablation of the other pulmonary veins, the left atrium posterior wall and the lateral mitral isthmus.

All ablation procedures were performed under general anesthesia and uninterrupted oral anticoagulation. After gaining vascular access, a diagnostic catheter was positioned in the coronary sinus. Fluoroscopy and transesophageal echocardiography guided, single transseptal puncture was performed to gain access to the left atrium. In cases in which an electroanatomic mapping system was used, a circular mapping catheter was roved through the atrium to generate a baseline voltage map of the left atrium, after which this mapping catheter was removed from the patient. If rotational angiography was used, the reconstruction of the left atrium was integrated in the electroanatomic mapping system. The ULTC ablation catheter was inserted in the left atrium in a straight form and then configured using the desired stylet [8] (typically 25 mm therapeutic loop, 10 mm pitch and 8 mm diagnostic loop). The esophageal warming balloon was positioned in the esophagus, guided by marker‐rings that are visible on fluoroscopy. The ablation catheter was positioned at the PV antrum (Figure 1a) and cryoablation energy was delivered (typically 30 or 60 s per energy application, followed by an equal thawing time), aiming for electrical block between the PV and the left atrium. For each catheter position that was potentially in the vicinity of the phrenic nerve (e.g., right PVs), a lower power freeze was applied to assess for any impairment of the phrenic nerve (“Cryomapping” [8]), after which the true ULTC application was delivered. Typically, an additional energy application of the same duration was delivered after each energy application (“Bonus freeze”). In addition to isolation of the PVs, operators could use the ULTC system to ablate non‐PV targets (Figure 1b,c), including the posterior wall of the left atrium (LAPW), the lateral mitral isthmus (LMI, mitral valve annulus to left inferior PV), and the cavotricuspid isthmus (CTI, tricuspid valve annulus to inferior vena cava). The LAPW was targeted by applying overlapping circular ablation lesions at the area between the PVs, aiming for electrical block between this area and the rest of the left atrium. CTI and LMI block were achieved using a dedicated stylets which facilitated linear ablation lesions across the isthmus. After ablation, the ablation lesions were assessed for electrical block using electrograms and pacing maneuvers or voltage mapping. In case an ablation target was not electrically isolated, additional ULTC applications could be administered until all lesions were electrically blocking.

### 2.3 Repeat Ablation

Repeat ablation procedures were performed to treat arrhythmia recurrence after ULTC. The timing and technique of the repeat ablation procedure were at the discretion of the treating physician. Repeat ablation procedures commenced with assessment of the ULTC ablation lesions. Evaluation of PV lesions included assessment of electrical block using pacing maneuvers, and assessment of electrical activity in the PVs using electrograms and electroanatomic mapping. Evaluation of LAPW lesions involved assessment of electrical activity at the area between the PVs, typically using electroanatomic mapping. Voltage maps were used to assess local voltage at the LAPW, and activation maps were used to assess any electrical potentials travelling across the LAPW. Finally, evaluation of LMI and CTI lesions was performed by assessing electrical block using pacing maneuvers, and by assessing electrical activity at the LMI or CTI using electroanatomic mapping. If a patient had a regular atrial tachyarrhythmia (e.g., atrial flutter or focal atrial tachycardia), the arrhythmia mechanism was assessed using activation maps. Reconnected ablation targets and other arrhythmia mechanisms were targeted with RFA until all target ablation lesions showed electrical block.

### 2.4 Follow‐Up After Ablation

There was no standardized follow‐up regimen to assess arrhythmia recurrence after the ablation procedures. Following the index ULTC procedure, follow‐up could be strictly protocolized if patients were enrolled in the CryoCure‐2 study or per institutional standards if patients were enrolled in the iCLAS Cryoablation System PMCF study. Protocolized follow‐up of CryoCure‐2 participants included clinical visits and 24‐h Holter monitoring at 3 and 6 months, and 7‐day Holter monitoring at 12 months [8]. Per institutional standards, patients were typically invited for outpatient clinic visits at 3‐ and 12‐months post ablation, which included rhythm assessment using a 12‐lead electrocardiogram and Holter monitoring in patients with recurrent symptoms after repeat ablation. Furthermore, patients were encouraged to acquire symptom driven electrocardiograms after repeat ablation. Follow‐up after repeat ablation was not strictly protocolized and was performed per institutional standards, as described above. Arrhythmia recurrence was defined as electrocardiographic documentation of atrial tachyarrhythmia following the ablation procedure, excluding a 3‐month blanking period.

### 2.5 Data Collection and Statistical Analysis

**Figure 2:**
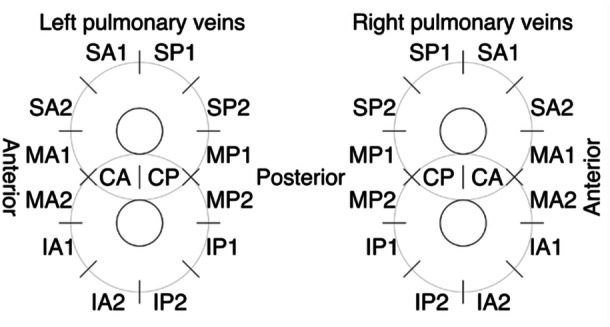
Segment based pulmonary vein gap assessment. A: Anterior, C: Carina, I: Inferior, M: Mid, P: Posterior, S: Superior.

**Figure 3:**
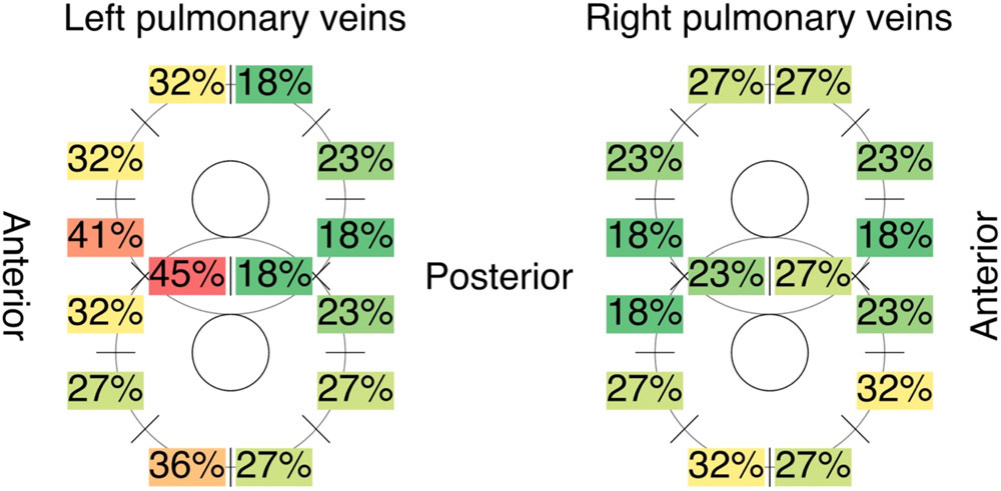
Segment based pulmonary vein ablation lesion assessment. Per segment, the percentage indicates the proportion of patients with reconnection of the ablation lesion segment.

Data on baseline and procedural characteristics, as well as arrhythmia outcomes after ablation were collected. For repeat ablation procedures, sites of PV reconnection were recorded in a segment‐based data collection sheet (Figure 2). Statistical analysis was performed using R (R Foundation for Statistical Computing, Vienna, Austria) using the following packages: tableone, tidyverse, ggpubr, ggthemes, survival & survminer. Figures 2 and 3 were created using Affinity Designer (Serif (Europe) Ltd., Nottingham, United Kingdom). Descriptive statistics were presented as counts and percentages for categorical variables, means and standard deviations for normally distributed continuous variables, and medians and interquartile ranges for non‐normally distributed continuous data. Arrhythmia recurrence after repeat ablation was visualized using a Kaplan‐Meier curve.

## 3 Results

### 3.1 Baseline Characteristics

Out of the 138 patients that had a ULTC ablation to treat AF between September 2018 and June 2023, 25 patients (18%) had a repeat ablation procedure, which were included in the study cohort. The baseline characteristics of the patients in the cohort (Supplementary Table 1), before the ULTC ablation procedure, are summarized in Table 1.

**Table 1:** Baseline characteristics.

	Cohort (*n* = 25)
Age, years	68.3 ± 7.1
Male sex	17 (68)
BMI, Kg/m^2^	28.2 ± 3.7
CHA_2_DS_2_‐VASC	
0	5 (20)
1	2 (8)
2+	18 (72)
OSAS	4 (16)
COPD	4 (16)
Atrial fibrillation	
paroxysmal	8 (32)
persistent	17 (68)
long‐standing persistent	0 (0)
Time since diagnosis of atrial fibrillation, years	4.2 [0.22–6.5]
LAVI, mL/m^2^	42.6 ± 24.4
LVEF, %	54 ± 9

*Note:* Normally distributed continuous data are presented as mean ± standard deviation. Non‐normally distributed continuous data are presented as median[interquartile range]. Categorical data are presented as count(percentage of total).

Abbreviations: BMI, body mass index; COPD, Chronic obstructive pulmonary disease; LAVI, Left atrial volume index; LVEF, Left ventricular ejection fraction; OSAS, Obstructive sleep apnea syndrome.

### 3.2 Ultra‐Low Temperature Cryoablation Index Procedure

At the start of the ULTC index procedure, 13/25 patients were in AF while the remaining were in sinus rhythm. An electroanatomic mapping system was used in 16/25 patients (CARTO (Biosense Webster, Diamond Bar, CA, USA) in 13 patients, KODEX‐EPD (EPD Solutions, a Philips company, Best, The Netherlands) in 2 patients, and EnSite (Abbott Cardiovascular, Plymouth, MN, USA) in 1 patient). The ULTC ablation system was used to isolate the PVs in all patients, the LAPW was targeted in 15 patients, the LMI was targeted in 5 patients and the CTI in 2 patients. The median number of ULTC applications and procedural times are presented in Table 2. No patients had common PV ostia or accessory PVs. One or more electrocardioversions were performed during the procedure in 12/25 patients. At the end of the procedure, all patients were in sinus rhythm.

**Table 2:** Procedural characteristics ULTC index procedure.

	Cohort (*n* = 25)
PV applications	
LSPV	2 [2–3]
LIPV	2 [2–3]
RSPV	2 [2–3]
RIPV	2 [2–2]
LAPW applications	6 [4–6]
LMI applications	6 [2–6]
CTI applications	4.5 [3.75–5.25]
ULTC ablation time, minutes	12.2 ± 4.8
Left atrium dwell time^a^, minutes	65.1 ± 34.8
Fluoroscopy time^b^, minutes	16.2 ± 6.4
DAP^c^, Gy·cm2	18.2 ± 18.5
Total procedure time, minutes	107.3 ± 43.7

*Note:* Normally distributed continuous data are presented as mean ± standard deviation. Non‐normally distributed continuous data are presented as median[interquartile range].

Abbreviations: CTI, cavotricuspid isthmus; DAP, dose area product; PV, pulmonary vein; LA, left atrium; LAPW, left atrium posterior wall; LIPV, left inferior PV; LMI, lateral mitral isthmus; LSPV, left superior PV; RIPV, right inferior PV; RSPV, right superior PV; ULTC, ultra‐low temperature cryoablation.

^a^ Available for 19/21 patients.

^b^ Available for 24/25.

^c^ Available for 23/25.

### 3.3 Repeat Ablation Procedure

Repeat ablation procedures were performed after a median of 12.1 [7.4–22.6] months post‐ULTC. An electroanatomic mapping system was used in all cases: Carto in 17 patients, EnSite in 7, and AcQMap(Acutus Medical, Carlsbad, CA, USA) in 1. Pacing maneuvers and electroanatomic mapping were used to assess electrical block of the ULTC ablation lesions (See: Ablation lesion durability) and the arrhythmia mechanisms of any regular atrial tachyarrhythmia that occurred during the procedure (See: Procedural Arrhythmia). Reconnected ablation targets and additional tachycardia mechanisms were targeted using RFA in all patients. After ablation of all targets, six patients (24%) remained in arrhythmia (all AF), which were successfully electrocardioverted to sinus rhythm. The procedural characteristics of the repeat ablation are summarized in Table 3.

**Table 3:** Procedural characteristics repeat ablation.

	Cohort (*n* = 25)
Incomplete PV lesion	
1 PV	3/25 (12)
2 PV	8/25 (32)
3 PV	4/25 (16)
4 PV	6/25 (24)
Incomplete LAPW lesion	4/15 (27)
Incomplete LMI lesion	3/5 (60)
Incomplete CTI lesion	1/2 (50)
Fluoroscopy time^a^, minutes	7.5 ± 6.6
DAP^a^, Gy·cm2	9.2 ± 14.4
Total procedure time^a^, minutes	90.0 ± 32.7

*Note:* Continuous data are presented as mean ± standard deviation. Categorical data are presented as count/total (percentage of total).

Abbreviations: CTI, cavotricuspid isthmus, DAP, dose area product; LAPW, left atrium posterior wall, LMI, lateral mitral isthmus, PV, pulmonary vein.

^a^ Available for 24/25 patients.

### 3.4 Ablation Lesion Durability

Reconnection was observed in 55/100 PVs, resulting in a median of 2 [1–3] reconnected PVs per patient (Table 3). This included 14/25 of left superior PVs, 16/25 left inferior PVs, 10/25 right superior PVs and 15/25 right inferior PVs (*p* = 0.34). Assessment of additional ablation lesions revealed that the posterior wall was reconnected in 4/15 patients, the CTI lesion in 1/2 patients and the lateral mitral isthmus lesion in 3/5 patients. Segment based ablation lesion data were available for 22/25 patients and are illustrated in Figure 3. There was a significant difference between the number of gaps when comparing the anterior and posterior halves of the left and right PV's(*p* = 0.047), with most reconnected segments in the anterior side of the left PVs (35% of segments), followed by the anterior side of the right PVs (25%), posterior side of the right PVs (24%), and lastly the posterior side of the left PVs (22%). The segment that was reconnected most often was the anterior part of the carina of the left PVs (‘CA’, 45%), followed by the adjacent segment on the anterior side of the left superior PV (‘MA1’, 41%) and the anteroinferior segment of the left inferior PV (‘IA2’, 36%).

### 3.5 Procedural Arrhythmia

The rhythm at the start of the repeat ablation procedure was sinus rhythm in 15 patients, AF in four patients and atrial flutter in six patients. During the repeat ablation procedures, atrial macroreentry (spontaneous or induced by programmed electrical stimulation) occurred in three other patients and AF in three other patients. The mechanisms for the atrial macroreentry were perimitral reentry (2 patients), CTI‐dependent reentry (1 patient), reentry around left atrial anterior fibrosis (2 patient), reentry around the ligament of Marshall (1 patient), reentry around left atrial posterior scar (2 patients) and reentry around septopulmonary bundle (1 patient).

### 3.6 Follow‐Up After Repeat Ablation

**Figure 4:**
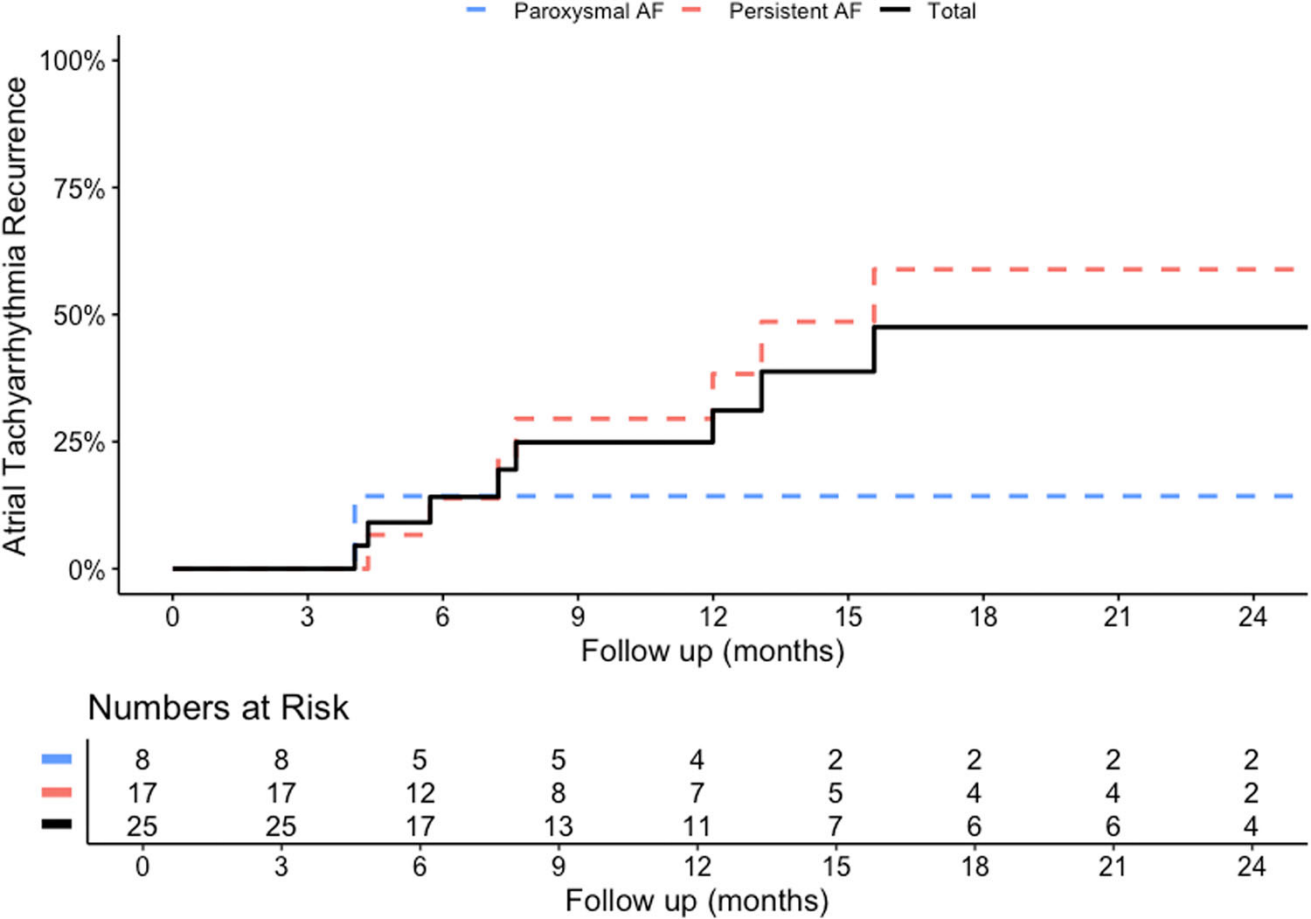
Kaplan‐Meier Curve illustrating recurrence of atrial tachyarrhythmia after repeat ablation. The black line illustrates the complete cohort. The dashed blue and red lines illustrate the patients that had paroxysmal and persistent atrial fibrillation before the ULTC ablation procedure.

The median follow‐up time after repeat ablation was 12.6 [7.6–28.2] months, with 15/25 patients having a follow‐up of over 1 year. At 1 year follow‐up, the Kaplan‐Meier estimate for atrial tachyarrhythmia recurrence rate was 31.1% (Figure 4). During the complete follow‐up time, 10/25 patients had documented recurrence of atrial tachyarrhythmia. This included AF in eight patients and atrial flutter in four patients. No cases of atrial tachycardia recurrence have been recorded. Of the patients without arrhythmia recurrence after repeat ablation, five patients were without anti arrhythmic drugs, three were treated with beta blockers, and seven with class 3 anti arrhythmic drugs. Of the patients with arrhythmia recurrence, three were treated without anti arrhythmic drugs, one was treated with beta blockers, two were treated with class 3 anti arrhythmic drugs, two had a repeat catheter ablation, and two had pacemaker implantation and His bundle ablation. Outcomes of repeat mapping are available for 1 of the 2 patients that had a repeat ablation procedure. At the ULTC procedure, the patient had PVI + LAPW ablation. During the repeat ablation procedure, gaps in both the PVs and LAPW lesion were targeted. During the second repeat ablation procedure, the PVs were isolated but the LAPW was targeted again. Additionally, a line was created along the anterior LA (RSPV to mitral valve annulus) to treat a perimitral flutter.

## 4 Discussion

This is the first study to provide insights in the findings and outcomes of repeat ablation after ULTC: Describing patterns of ablation lesion reconnection after ULTC and arrhythmia recurrence rates after repeat ablation with a median follow up time of over 12 months.

The main findings of this study include: (I) Reconnection of one or more PV was observed in 84% of patients that had a repeat ablation for arrhythmia recurrence after ULTC ablation. (II) Reconnection occurred most often at the anterior part of the left PVs. (III) Assessing additional ablation lesions, LAPW isolation was incomplete in 4/15 patients (27%), CTI lesions were reconnected in 1 out of 2 patients (50%) and LMI lesions in 3/5 patients (60%). (IV) After ULTC ablation and repeat ablation using RFA, 10 out of 25 patients (40%) had arrhythmia recurrence after a median follow‐up of 12.6 [7.6–28.2] months.

### 4.1 Pulmonary Vein Reconnection

Clinicians could expect that the ultra‐low temperatures achieved by this system would guarantee durable ablation lesions, even in thicker myocardial structures like the LSPV‐LAA ridge. However, in this cohort, 84% of patients undergoing a repeat ablation procedure to treat arrhythmia recurrence after ULTC had reconnection of one or more PVs. This is in line with conventional thermal ablation modalities, as PV reconnection was observed in 69%–100% after CBA and 80%–100% after RFA [3, 4, 9, 10, 11, 12, 13]. Likewise, the proportion of reconnected veins was 55% after ULTC, while this was 20%–52% for CBA and 36%–67% for RFA [3, 4, 9, 11, 12, 13]. Contrastingly, PV reconnections seem to occur less frequently after PFA, as reconnections were observed in only 58%–62% of patients and 29%–31% of veins that were treated with PFA using a pentaspline ablation catheter (FARAPULSE, Boston Scientific, Marlborough, USA) [14, 15]. This suggests that PV reconnection is an important driver for arrhythmia recurrence after both ULTC and conventional thermal ablation modalities, while it could be of less importance in arrhythmia recurrence after PFA, in which other mechanisms could be of greater importance (e.g., sparing of the ganglionated plexi [16, 17]). Although it appears that PV reconnection is a shared mechanism of arrhythmia recurrence for thermal ablation modalities, the pattern of PV reconnection differs. For CBA, the RIPV is reconnected most often, which could be due to insufficient catheter‐tissue contact as a result of imperfect alignment of the CBA catheter with the PV [13]. On the other hand, the anterior part of the left PVs appears to be reconnected most often after RFA, which could be the result of greater myocardial thickness of the ridge between the left superior PV and the left atrial appendage ridge, and ligament of Marshall [18]. In the present study, the location of PV reconnection was not preferential to a specific PV. However, the anterior side of the left PVs seemed to be reconnected more often. This location of reconnection could be the result of the thicker myocardium in this area that may hinder the creation of durable ablation lesion formation, despite the extreme temperatures that are applied with ULTC. Positioning may also be less optimal, with insufficient adhesion to the ridge or partial positioning over left atrial appendage orifice that would leave a gap. These findings could affect ULTC energy application strategy, as operators could place more focus on the energy applications at the anterior part of the left PVs (e.g., ensure optimal catheter‐tissue contact, apply ULTC energy for a longer duration, or apply multiple ULTC applications). Additionally, a hybrid ablation modality of ULTC and PFA is currently under investigation which may promote more effective isolation of the PVs while also modulating the autonomic nerve system (PARALELL trial, NCT05408754) [19].

### 4.2 Additional Ablation Lesions

Although multiple studies have been conducted to assess the efficacy of catheter ablation beyond PVI, no lesion sets have consistently shown additional benefit. In contrast, more extensive lesion sets have been applied in surgical ablation procedures with high rates of success. Possibly, the presence of nondurable ablation lesions could explain the difference in efficacy of extra‐PV ablation lesions. As ULTC holds the promise of creating durable ablation lesions and the stylet‐based platform of the ULTC ablation catheter was designed with flexibility of ablation lesions in mind [20], the ULTC system could theoretically be used to enable durable ablation lesion creation outside the PVs. As extra‐PV ablation lesions were also applied in the patients in this cohort, we also assessed the durability of these extra‐PV ablation lesions.

The lesion that was targeted most often in addition to the PV's was isolation of the LAPW. In this study, 4 out of 15 patients (27%) with initial LAPW isolation had reconnection of the posterior wall. This number was relatively low compared to the 40%–46% reconnection rate that was observed in studies on LAPW isolation using RFA [21, 22]. Contrastingly, the reconnection rate was relatively high compared to the rates of LAPW isolation using CBA (17.3% [23]) and PFA (15% [24]). Following isolation of the LAPW, ablation of the LMI was performed the second most often. In prior studies that used RFA to target the LMI, reconnection was found in 62%–90% of patients [25, 26, 27, 28]. These reconnection rates can be considered similar or relatively high compared to the 60% rate of this cohort. However, it must be noted that the number of patients in this study was relatively low (LMI ablation performed in only 5 patients). Lastly, CTI ablation was performed in two patients of the cohort. Reconnection was found in 1 of these two. This was generally in line with prior studies in which RFA was used to target the CTI, as reconnection rates of up to 39% were found for CTI ablation alone [29] and 52% for CTI ablation as an adjunct to PVI [30].

### 4.3 Recurrence After Successful Repeat Ablation

Despite the repeat ablation procedure, there was arrhythmia recurrence in this cohort. The 1‐year Kaplan‐Meier estimate for arrhythmia recurrence after repeat ablation was 31.1%. This was higher than the patients with repeat ablation in the FIRE AND ICE trial, which showed 1‐year recurrence rates of 18.2% and 15.6% for CBA and RFA, respectively [12]. A potential explanation for this difference is the high rate of persistent AF patients included in the present study, as patients with persistent AF typically have more advanced arrhythmic substrate. This is illustrated in another study on repeat ablation after RFA or CBA that included 63% non‐paroxysmal AF, in which the 1‐year recurrence rate was approximately 36%, which is in the same range as the present study [3].

### 4.4 Limitations

This was a retrospective analysis of patients that were enrolled in two prospective observational studies on ULTC. This may have caused heterogeneity in the cohort, as patient selection, ablation strategy (e.g. ULTC application duration, applied lesion sets, use of electroanatomic mapping systems, etc.) and follow‐up regimens varied among patients. For example, the duration of ULTC applications was determined based on the ‘time to effect’ in patients that were enrolled in the CryoCure 2 study [6], while it was typically more pragmatic (i.e., 30 or 60 s per application) in patients in the iCLAS PMCF study. Due to the retrospective nature of this study, the total energy application duration per ablation target was missing in some patients, yielding it impossible to assess whether ULTC application duration could be associated with reconnection of an ablation target. An additional limitation of the retrospective design of this study is the non‐protocolized follow‐up regimen after repeat ablation. Therefore, the arrhythmia outcomes after repeat ablation could be underestimated, as mainly symptomatic rhythm monitoring was performed. Lastly, this was a multicenter study that included two centers with very experienced ULTC operators, which may limit the generalizability of outcomes.

## 5 Conclusion

The mechanism of arrhythmia recurrence after ULTC appears to be similar to conventional thermal ablation modalities, as rates of PV and LAPW recurrence rates are comparable. Notably, the anterior part of the left PVs appears to be reconnected most often, highlighting that this area is susceptible to result in nondurable ablation lesions.

## Ethics Statement

All subjects provided informed consent for study participation. The study was conducted in accordance with the Declaration of Helsinki, and the protocol was approved by the ethics committee and local institutional review board.

## Conflicts of Interest

Dr. De Potter has received institutional support from Adagio Medical; Dr. Boersma reports that the Cardiology Department received consulting fees from Adagio Medical; Drs. Abeln and Drs. Addeo have no conflicts of interest.

## Supporting information

SuppTable_1.

## Data Availability

The data that support the findings of this study are available upon reasonable request.
