# Supplementary material for: Durability of Ultra‐Low Temperature Cryoablation Lesions in Atrial Fibrillation: Insights From Repeat Ablation Procedures
Source: J Cardiovasc Electrophysiol. 2025 Mar 27;36(6):1264–71. doi: 10.1111/jce.16665 (PMC12160705; doi:10.1111/jce.16665)
Supplement: Supplementary file 1 — SuppTable_1. [file JCE-36-1264-s001.docx]

| **AF type** | **AF Duration** | **LA volume** | **Lesions in addition to PVI** | **Arrhythmia Recurrence** | **Reconnected ablation lesions** |
| --- | --- | --- | --- | --- | --- |
| paroxysmal | 0.2 | 21.9 | None | Paroxysmal AF | LSPV, LIPV, RSPV, RIPV |
| paroxysmal | 0.2 | 32.5 | None | Persistent AF & Regular SVT | None |
| paroxysmal | 0.2 | 29.3 | None | Regular SVT | LSPV |
| paroxysmal | 0.6 | 29.1 | None | Paroxysmal AF | LSPV, LIPV, RSPV, RIPV |
| paroxysmal | 2.3 |  | None | Regular SVT | LSPV, LIPV, RIPV |
| paroxysmal | 6.5 | 26.0 | None | Paroxysmal AF | RIPV |
| paroxysmal | 9.8 | 40.0 | None | Paroxysmal AF | LIPV, RIPV |
| paroxysmal | 10.7 | 40.0 | None | Paroxysmal AF | LIPV, RIPV |
| persistent | 0.1 | 39.0 | None | Persistent AF | LSPV, LIPV, RSPV |
| persistent | 0.1 | 44.0 | None | Persistent AF & Regular SVT | LSPV, RIPV |
| persistent | 0.1 | 24.6 | LAPW | Regular SVT | LSPV, LIPV, RSPV, RIPV, LAPW |
| persistent | 4.2 | 42.1 | LAPW | Persistent AF | LSPV, LIPV |
| persistent | 5.0 | 38.0 | LAPW | Persistent AF | LIPV, RSPV, RIPV, LAPW |
| persistent | 5.7 | 28.4 | LAPW | Regular SVT | RIPV |
| persistent | 6.3 | 125.0 | LAPW | Persistent AF & Regular SVT | LSPV, LIPV |
| persistent | 7.5 | 42.7 | LAPW | Persistent AF | LSPV, LIPV |
| persistent | 7.7 | 54.7 | LAPW | Persistent AF | None |
| persistent | 9.1 | 30.0 | LAPW | Paroxysmal AF | LIPV, RSPV, RIPV, LAPW |
| persistent | 15.2 | 35.8 | LAPW | Persistent AF | RSPV, RIPV |
| persistent | 0.0 | 55.0 | LAPW+LMI | Paroxysmal AF | LSPV, LIPV, RSPV, RIPV, LMI |
| persistent | 0.2 | 31.1 | LAPW+LMI | Regular SVT | LSPV, LIPV, RSPV, RIPV |
| persistent | 0.4 | 37.0 | LAPW+LMI | Persistent AF & Regular SVT | None |
| persistent | 5.9 | 30.5 | LAPW+LMI | Persistent AF | LSPV, LIPV, LMI |
| persistent | 5.4 | 108.6 | LAPW+CTI | Persistent AF | LAPW |
| persistent | 2.8 | 37.5 | LAPW+LMI+CTI | Regular SVT | LSPV, LIPV, RSPV, RIPV, LMI, CTI |

Supplementary Table 1. Study population

AF duration is presented in years. LA volume is presented in mL/m^2^. Abbreviations: AF: Atrial fibrillatiom, LA: Left atrium, PVI: Pulmonary Vein Isolation, SVT: Supraventricular tachycardia.
